# 

**Published:** 1886-05

**Authors:** 


					﻿MAY, 1886-
EtChft OlrfChONAL PAGES
■QtriSERIES
Vol. XXV
NEW SERIES
Vol. III.—No. 3-\ ;


Wedical^^K
Journal
"WOO?
i^ax et	toore •}

(Gjtau
1 W.F.WESTMORELlAND.Mfe..
H.V.M.M1LLER.M.D.LL .D.®
JAMES A.GRAY. M.D.
WtlBLlSHEO BY JAMES PHARRISON&.GK
Atlanta,GA. £A
onnscoiPTION! 12.BO A YEAR.
				

